# Geopolymers Manufactured by the Alkali Activation of Mining and Ceramic Wastes Using a Potential Sustainable Activator from Olive Stone Bottom Ashes

**DOI:** 10.3390/ma18030688

**Published:** 2025-02-04

**Authors:** Raul Carrillo Beltran, Elena Picazo Camilo, Griselda Perea Toledo, Francisco Antonio Corpas Iglesias

**Affiliations:** Research Group TEP 222 “Materials and Mining Engineering”, Higher Polytechnic School of Linares, University of Jaen, 23700 Linares, Jaen, Spain; epicazo@ujaen.es (E.P.C.); gept0001@red.ujaen.es (G.P.T.); facorpas@ujaen.es (F.A.C.I.)

**Keywords:** geopolymer, slate stone cutting sludge, chamotte, olive biomass bottom ash, sustainable construction, valorization, circular economy

## Abstract

The reuse of by-products as alternative raw materials to traditional construction materials is required in order to ensure sustainable development in the construction sector and is a significant and important focus in the fields of materials science. This study developed geopolymers using by-products from mining, ceramics, and olive industries, including slate stone cutting sludge (SSCS) and chamotte (CH) as aluminosilicate sources, and olive biomass bottom ash (OSBA) as an alkaline activator with sodium silicate. A key novelty of the research lies in the use of SSCS, an underexplored by-product in geopolymerization studies, as a viable aluminosilicate source. The geopolymers were prepared with varying weight ratios of SSCS, CH, and OSBA/Na₂SiO₃ (1.7, 1.9, 2.2, and 2.4). Physical and mechanical tests determined the optimal formulation, while FTIR and SEM analyses revealed the material’s chemical and structural evolution. The FTIR analysis detected the quartz and carbonate phases, indicating incomplete quartz dissolution and carbonate formation during calcination. The SEM analysis revealed a dense microstructure with reduced porosity and enhanced geopolymerization in samples with higher OSBA content. The optimal geopolymer (60% OSBA, 30% CH, OSBA/Na₂SiO₃ ratio of 2.2) achieved a compressive strength of 33.1 MPa after 28 days. These findings demonstrate the feasibility of producing geopolymers using SSCS, CH, and OSBA, promoting the reuse of industrial by-products as sustainable alternatives to conventional binders.

## 1. Introduction

The principles of sustainable development in construction [1] require that the building and construction sector be as green as possible, reducing gross annual carbon emissions worldwide [2] and the massive consumption of natural resources for construction materials. Both sectors extensively use ordinary Portland cement (hereinafter referred to as OPC) which leads to adverse effects, including, as follows: (i) GHGs emissions emitted in the manufacturing of OPC account for 7% of gross annual CO_2_ emissions [3]; and (ii) shortages of raw materials (taking into consideration that 1.50 t of raw materials are needed for 1 ton of OPC) [4].

With the aim of reducing the CO_2_ emitted in the manufacturing process, the field of materials engineering is taking steps to reduce the consumption of non-renewable materials and improve the performance of building and construction materials. To this end, the materials engineering field has identified potential strategies to make the OPC sector more sustainable through the replacement of OPC with other materials, including, as follows: low-carbon supplement cementitious materials; cements with a higher proportion of pozzolanic and hydraulic additions [5,6,7]; low-carbon binders, calcium sulfoaluminate cements and calcium cements [8,9,10,11,12]; magnesia cements [13,14,15]; belite Portland cements, and geopolymer binders [16]. Geopolymers, as alternative materials, have been of great interest for the research community as these possess environmental advantages in comparison to other cementitious materials [17]. Geopolymers, also known as alkali-activated materials, are inorganic polymers generated by the combined reaction of a source of aluminosilicates, as a solid phase, with an alkaline activator as a liquid phase that is rich in alkaline ions [18,19,20]. This technology can be considered sustainable due to the recycling of different by-products rich in silica and reactive alumina [21] that may trigger several chemical reactions, leading to an amorphous aluminosilicate network [22]. These binders offer the possibility of stabilizing inert mining wastes in the geopolymer matrix and reducing greenhouse emissions and energy consumption due to the relatively low temperatures required for synthesis (below 100 °C) [23,24]. Therefore, geopolymers are a potential solution for the correct management of uncontrolled waste in landfill. Previous researchers have studied the development of geopolymers through the valorization of agricultural, industrial, mining, and human-made wastes such as biomass ash [25,26,27]; slag [24,28,29]; chamotte and red ceramics [18,30]; cutting sludges and tailings from mining [31,32,33,34,35]; construction and demolition materials [16,36,37,38]; and glass from urban waste [39,40]. Due to the different sources of aluminosilicates, studies have shown that the final properties and characteristics of a geopolymer, such as low thermal conductivity [23], fire resistance [41], acid attack resistance [42], and compressive strength [24], are directly dependent on the types of raw materials used in the geopolymerization process.

In addition to the significant advantage of reusing different by-products as precursors in the geopolymerization process, geopolymers can be also obtained from the valorization of by-products [43,44,45] leading to similar mechanical and crystalline phases as those obtained with conventional activators [18,43,45]. The fact that the alkali activation can be partially or fully prepared using by-products makes them a viable alternative to commercial alkaline solutions. Industrial chemical activators, such as sodium hydroxide, potassium hydroxide, and silicates, are leading to increases in both greenhouse emissions (hereinafter referred to as GHGs) [46] and energy consumption for their manufacturing [47], and to increases in the associated costs of the final product [48,49].

Based on the abovementioned factors, geopolymerization technology was selected as a sustainable approach in this study. Three by-products from the ceramics, mining, and biomass power-plant industries were chosen as raw materials; their suitability and homogeneity for the geopolymerization process were explored.

The experimental geopolymer consisted of a combination of raw materials from a slate stone mining site in La Cabrera Baja Region (Leon, Spain); olive biomass bottom ash from the calcination of olive stone (OS) waste from olive oil extraction processing, collected from a power plant in Linares (Jaen, Spain); and chamotte obtained from a ceramic brick industry located in Bailen (Jaen, Spain). By-products from the mining and ceramics industry were used as source of aluminosilicates, and olive biomass bottom ash, along with sodium silicate and distilled water, was used for the alkali activation solution preparation.

Previous studies [50,51,52,53,54,55] have demonstrated that slate stone has a high percentage of alumina and silica in its composition; therefore, slate stone cutting sludge (hereinafter referred to as SSCS) is a potential source of aluminosilicates for geopolymers. Chamotte (hereinafter referred to as CH) has been studied as a suitable precursor for the synthesis of geopolymers due to its aluminosilicate contents [18,30,43]. Under alkaline conditions, aluminosilicates undergo depolymerization and structural reconfiguration, forming Si-O-Al bonds that generate a three-dimensional amorphous matrix. This in situ polymerization significantly influences the mechanical properties of the resulting material. Previous studies have shown that an optimal Si/Al molar ratio in geopolymerization reaction precursor materials improves mechanical strength by promoting the formation of a denser and more homogeneous aluminosilicate gel [56,57,58]. Chamotte is also characterized by pozzolanic activity [59,60]. This research program also investigated the valorization of olive biomass bottom ash, from the calcination of olive stone (hereinafter referred to as OSBA) as a residue of olive oil production, because the ash is mainly composed of potassium and can thus be used for the preparation of an alkali activator solution.

Apart from the research program studying the suitability of raw materials for geopolymer conformation, another equally and significant aim of this study is to offer sustainable solution for the recovery of waste that is currently accumulated in large quantities, with subsequent negative impacts on the environment both in terms of contamination, biodiversity impact and land use.

In Spain, the mining industry, which extracts and processes slate, has generated large quantities of mining tailings and slate stone cutting sludge over the years, especially the latter, to meet international demands [10,12]. The northern areas of Spain, and more specifically the regions of Galicia, and Castilla and Leon, contain a significant volume of slate mining waste in various forms.

In the ceramics industry, the accumulation of broken or defective fired clay bricks, that do not meet visual or market requirements, is also a problem in terms of managing the large volume of waste. Once sintered, this by-product cannot be reintroduced into the brick-making process. Storage of these wastes has resulted in environmental impacts. Other techniques for the valorization for the chamotte include its reuse as a granular fill material for roads or in formulations of refractory concretes, among others.

In this experiment, chamotte was used to replace metakaolin in the formulation of the geopolymer samples. Previous studies have shown that chamotte presents lower reactivity than metakaolin [58]; its use as an addition for reinforcing final geopolymer properties has thus been recommended.

As mentioned above, this research program also explores the valorization of olive biomass bottom ash from the calcination of olive stone (OS) wastes from the olive oil extraction process. The ash is abundant, as the olive biomass is used for the generation of electricity. The ash is mainly composed of potassium and can be used for the alkali activator solution preparation.

In recent years, the research community has investigated the addition of combustion ash from different sources [15,16,17,18,19] into ceramic bricks [30], and for cementitious products [59]. The olive industry generates large amounts of different by-products such as stones, leaves, and direct waste from pruning activities. The olive biomass is currently used as fuel for power generation. Disposal in landfills of this biomass waste material has impacts on the environment and is costly for power plants.

Based on the potential opportunities that geopolymer technology brings through the valorization of by-products from different sources in precursor and alkali activation. The aim of this research is threefold; firstly to study this technology as a promising sustainable solution; secondly a mitigation measure for the uncontrolled wastes from mining, power plant biomass, and ceramic industries; and thirdly the development of sustainable new alternative binder materials. This work focuses on the development of a geopolymeric mortar on a laboratory scale; therefore, a cost analysis for large-scale production was not considered, in principle. However, the main raw materials (SSCS, CH and OSBA) are industrial, mining, and agricultural by-products, most of which are obtained at low or even no cost, as they represent waste generated by other industries. This aspect significantly reduces the initial costs compared to the use of virgin materials. To obtain a first order of magnitude regarding the factors influencing production costs and as a preliminary cost estimation, energy consumption for the curing process and the contributions of each main component in the final geopolymer were considered. In terms of energy consumption for the curing process used in this research, guided by data from previous studies [60,61], the baseline amount of energy for 90 h of curing at 85 ± 5 °C h is estimated at around 6 KWh. From the standpoint of contributions from the components and production process to GHG emissions (kg CO_2_e/m^3^), the use of alkali solutions of sodium hydroxide represents most of the total cost of the geopolymer [60], with an approximate value of 250 kg CO_2_e/m^3^ of GHG emissions, which is less in comparison to that for OPC (400 kg CO_2_e/m^3^). Therefore, the development of sustainable alkaline activators, through the reuse of ash, could reduce production costs and develop new lines of research.

To ensure that the research targets were achieved, raw materials were tested through a typical chemical and physical characterization test campaign. Different geopolymer samples were prepared using combinations of SSCS and CH as the source of aluminosilicates, and sodium silicate and OSBA as the alkali activation solution. The geopolymer samples were then subjected to physical and mechanical tests.

## 2. Materials and Methods

The following section describes the selected raw materials used to prepare the geopolymer specimens, and the applied experimental methodology for determining their suitability for the alkali activation process.

### 2.1. Raw Materials

The raw materials used in this experimental program are by-products from different sources: Slate stone cutting sludge and chamotte were the sources of aluminosilicates, and olive biomass bottom ash was used for the alkaline activator solution in combination with sodium silicate. The control geopolymer was prepared using sodium hydroxide and sodium silicate.

#### 2.1.1. Source of Aluminosilicates: Slate Stone Cutting Sludge from La Cabrera Baja (Leon, Spain) and Chamotte from Bailen (Jaen, Spain)

La Cabrera Baja, in the Spanish province of Leon, is characterized by a strong mining tradition of slate stone, with numerous mining sites and associated processing factories. The main mining wastes generated are sludges from operations to extract and cut the slate into smaller blocks and pieces for commercialization. The use of water saw systems lead to slate stone cutting sludges, with a fine granulometry, which are accumulated in ponds. One of the main environmental issues related to this cutting sludge is the presence of heavy metals particles. caused by the mining tooling during the cutting and processing activities. These are carried out using water on the mine surface; particles are then dispersed into aquifers. Previous engineering mining studies concluded that the extraction and processing operations for mining slate only provides a yield ranging between 4 to 8%, taking into account that the production of slate mining waste is an average of 95% [59]. Therefore, the landscape alteration and contamination of aquifers and rivers by dragged sludges are the most worrying environmental problems for this type of mining.

Following a previous study that used slate stone cutting sludge samples from La Cabrera Baja (Leon), samples were dried in an oven at 105 ± 2 °C for 24 h to remove the humidity, and afterwards dried at room temperature. The mining raw material samples did not require any mechanical grinding to achieve a unform size distribution, as after they were dried, they became a dry grey finer powder.

Chamotte, also known as grog, which is produced as a result of the crushing and pulverization of bricks or other fired ceramic products, was collected from Baiceram, a ceramic industry in Bailen, in the Andalusian Province of Jaen. This was chosen due to previous satisfactory studies on geopolymer conformation and due to its high aluminosilicates contents [29,60]. For this research program, the chamotte was crushed and ground to yield a powder with a particle size of 250 μm sieve, for securing the proper homogenization with other raw materials and a better reactivity [61,62].

#### 2.1.2. Alkali Activation Solution: Olive Biomass Bottom Ashes from Power Plant in Linares (Jaen, Spain), Sodium Hydroxide, and Sodium Silicate

The raw material used for the alkali activation solution was bottom ash from olive stones used as biomass for energy generation carried out in the olive biomass waste-to-energy plant “Bioelectrica de Linares, Valoriza Group” located in the Andalusian city of Linares. The OSBA used in this research belongs to the by-product resulting from the combustion of olive prunings, leaves, and stones for the generation of electrical energy. It should be noted that the initial purpose of the investigation was to develop a sustainable geopolymer fully prepared with by-products; however, during the conformation of samples it was noted that a percentage of sodium silicate was needed to improve the workability and cohesion of the materials. This may be due to the fact that bottom ash is less reactive and therefore has a lesser degree of geopolymerization in comparison with fly ash [62,63,64]. Differences include a larger particle size, a lower specific surface area, and a higher content of inert impurities, which reduces the amount of aluminosilicate phases available for geopolymerization. These properties decrease the ability of OSBA to interact efficiently with alkaline solutions, resulting in a relatively low reactivity compared to that of fly ash. To overcome these challenges, different methods were evaluated in the literature. Among these, mechanical milling stood out as an effective strategy to reduce the particle size, thereby increasing its specific surface area and improving the interaction with the alkaline solution [65].

Additionally, heat treatments at moderate temperatures (700-1000 °C) can induce higher amorphousness in OSBA, increasing the availability of reactive silicon and aluminum species [66]. Hybrid blends combining OSBA with other more reactive materials, such as chamotte or metakaolin, have also been explored, contributing to the formation of more homogeneous aluminosilicate products [67,68,69,70].

Therefore, two types of alkali activation were prepared, as follows: (i) a solution of NaOH, Na_2_SiO_3_, and distilled water for the control geopolymer samples; and (b) a solution of OSBA, Na_2_SiO_3_, and distilled water for the geopolymer target samples.

The OSBA samples used in this experiment were subjected to a complementary calcination process at a high temperature of around 950 °C for 1 h, to avoid the incomplete combustion of ashes or too short a time of exposition. Once the OSBA sample was cooled, it was then passed through a sieve to achieve a uniform micron granulometry.

### 2.2. Methods

The aim of the methodology used in this experimental research program was to study the viability of a novel geopolymer binder that combines three different by-products as follows: slate stone cutting sludge and chamotte, as precursor materials, and an alkali activation mixture prepared with olive biomass bottom ash, sodium silicate, and distilled water.

The research program was split into chemical and physical characterizations of the raw materials from the biomass power plant, ceramics, and mining sectors, and physical and mechanical tests on the different geopolymer specimens containing different formulations.

In the first experimental phase, the three different raw materials were subjected to elemental analysis, particle size distribution, X-ray diffraction, X-ray fluorescence, real density, pH determination, and Fourier transform infrared spectroscopy (FTIR); these were used to analyze their properties and evaluate their suitability for use as raw materials in geopolymer conformation.

The second research stage consisted of physical and mechanical tests for the different geopolymer families. To that end, the research program aimed to study geopolymer samples consisting of constant amounts of SSCS and CH, and different OSBA/Na_2_SiO_3_ weight ratios (1.7, 1.9, 2.2, and 2.4). The different families of geopolymer samples were studied to understand their physical and mechanical properties and to determine a balanced suitable formula with an optimal combination of the precursor and alkali activation solution. For the curing process of the geopolymers, a two-stage approach was used: (i) first, the samples were cured at an ambient temperature of 25 ± 2 °C for 72 h.; and (ii) the samples were then placed in an oven at 85 ± 5 °C and cured for 90 h, and then cured again for 6 h at an ambient temperature, with a total curing process of 7 days.

The two-stage curing process was designed to optimize the physical and mechanical properties of the geopolymers by influencing polymerization reactions and microstructure development.

During the first stage, the room-temperature curing facilitated initial gelation, which is essential for the formation of the three-dimensional structure of the geopolymer. At this stage, the dissolved silicon and aluminum species begin to react, forming an amorphous aluminosilicate gel. This period is critical to ensure an even distribution of the reaction products and to prevent the formation of cracks due to sudden contractions.

The second stage, at a temperature of 85 °C, accelerates the polymerization reactions and promotes the development of a more crystalline microstructure. The increase in temperature benefits the mobility of ions, resulting in a higher densification of the matrix. This heat treatment also contributes to improving the mechanical strength and durability of the material by removing non-structural water and consolidating the internal structure of the geopolymer.

Finally, controlled cooling allows for the stabilization of the microstructure formed during the thermal process, avoiding internal stresses or cracks due to rapid temperature changes.

Finally, the geopolymer samples were demolded and then physically tested. The physical tests were carried out to identify phenomena, such as linear shrinkage and weight loss, that can occur during the geopolymerization process. Variations in weight and dimensions, after the curing process, are discriminatory requirements for construction materials but also key considerations in a sustainable approach for the specific reuse of these different by-products. The influence of water on the novel geopolymer samples was also evaluated through capillarity water absorption and cold-water absorption tests. These standardized tests were used to determine the capacity for absorbing water, and thus the mass variation. Bulk density, open porosity, and compressive strength tests were carried out to evaluate the mechanical properties of the geopolymer samples for comparison with other construction materials.

The standards followed in the applied methodology of this experimental program were, as follows: (i) mass loss (UNE-EN 13581:2003 Standard) [71]; (ii) linear shrinkage (UNE-EN 13872:2004 Standard) [72]; (iii) capillary water absorption (UNE-EN 1015-18:2003 Standard) [73]; (iv) bulk density and open porosity (UNE-EN 1015-10:2000 Standard) [74]; and (v) compressive strength (UNE-EN 1015-10:2020 Standard) [75]. Finally, the geopolymer samples were subjected to Fourier transform infrared spectroscopy (FTIR) and scanning electron microscopy (SEM).

#### 2.2.1. Chemical and Physical Characterization Methodology of the Different Raw Materials

The physical and chemical characterization tests of the used raw materials, SSCS, CH, and OSBA, constitutes a key phase in this experimental program for evaluating the feasibility of SSCS and CH as sources of aluminosilicates, and OSBA as a source for the alkali solution preparation. Key factors, such as chemical composition, particle surface area, and crystalline phases, were analyzed to understand the reactivity of different raw materials, which is an important parameter for precursors [47]. The precursor reactivity with the alkali activation solution is directly related to its high contents in silica and alumina, high surface particle area, and greater content of amorphous phases.

First, the physical characterization phase comprised a study of the grain sizes of SSCS, CH, and OSBA, in order to determine if the particle sizes were suitable for securing the high activation of precursor raw materials during the geopolymerization process. The laser particle size distribution test was carried out as per UNE-EN 933-1:2012 [76], using a Malvern Mastersizer Bruker D8 Venture (Malvern Instruments Ltd., Malvern, United Kingdom). The particle densities of the raw material samples were obtained using the pycnometer method, following the UNE-EN-1097-7:2009 standard [77].

Since the kinetics of the chemical reactions between aluminosilicates and the alkaline solution is also a crucial parameter, the three pH values were determined, following the UNE-EN 10390:2022 Standard [78].

Second, the chemical characterization stage consisted of analyzing the mineralogical and chemical compositions of the mining, ceramic, and power generation biomass wastes. Analyses of the percentages of carbon, hydrogen, nitrogen, and sulfur were performed using the CHNS analyzer (Leco TruSpec Micro Model, St. Joseph, MI, USA). The presence of volatile elements in the sample was determined by the loss on ignition method, which measured mass losses at a temperature of 950 ± 5 °C. The chemical compositions of the SSCS, CH, and OBBA samples were determined by X-ray fluorescence (XRF) using the Bruker M4 Tornado spectrometer (Bruker: Billerica, MA, USA). X-ray diffraction (XRD) analyses were obtained using the diffractometer model Bruker D8 Venture (Bruker: Billerica, MA, USA) in order to characterize the crystalline structures of the different raw material samples.

#### 2.2.2. Preparation of Novel Geopolymers Samples with Slate Stone Cutting Sludges, Chamotte, and Olive Biomass Bottom Ashes as Sustainable Raw Materials

In this section, the physical and chemical characteristics of the conformed geopolymers were determined through a tests campaign. For that purpose, different geopolymer samples were elaborated with different formulations, aiming to obtain the right combination of an aluminosilicate sources, SSCS and CH, and alkali activation solution, a combination of OSBA, distilled water, and sodium silicate.

For this phase, two types of geopolymer mixtures were prepared (Table 1) as follows: (i) seven specimens of control geopolymers formed by the mixture of SSCS, as precursor, and sodium hydroxide and sodium silicate with Na_2_SiO_3_/NaOH weight ratio of 3 considering a 12 M solution; (ii) and four families of novel geopolymer samples, with seven specimens each, prepared with SSCS and CH, with different weight percentages of Na_2_SiO_3_ and OSBA. The different OSBA/Na_2_SiO_3_ weight ratios considered for this study were 1.7, 1.9, 2.2, and 2.4. In the first mixture formulation, the sodium hydroxide plays a vital role for enhancing a proper dissolution of the precursor, while the sodium silicate serves as a dispersant agent for sodium hydroxide and as a plasticizer in the activated material sample.

For the preparation of both mixtures—the control geopolymer and the novel geopolymer—for the different families, the same methodology was applied to further evaluate the physical and chemical characterizations of the different geopolymer specimens.

Prior to the preparation of the two types of geopolymer families—the control geopolymer and novel geopolymers with combinations of SSCS, CH, and sodium silicate—the precursor raw materials, SSCS, and CH, were dried in an oven at 105 ± 2 °C for 24 h to remove possible moisture, due to both landfills being outdoors. Subsequently, the CH was crushed and sieved to yield a powder with a suitable particle size distribution for passing through a 0.25 mm sieve. The mining by-product from the slate stone cutting sludge ponds did not require grinding due to the powdery consistency caused by sieving. However, The mining by-product from the slate stone cutting sludge ponds did not require grinding due to the powdery consistency caused by sieving. However, the mining waste was sieved to ensure 0.25 mm particle size. For precursor raw materials, finer particles improve the homogenization between the particles of the materials, and result in a better reactivity and higher compressive strength of the final geopolymer.

For the control geopolymer, the alkali solution, formed by sodium hydroxide and sodium silicate, was prepared in advance and cooled for 24 h at room temperature due to the exothermic reaction before being combined with SSCS following the proportions indicated in Table 1. In the particular case of the targeted novel geopolymer, for the alkali solution preparation, the calcined OSBA was also crushed to obtain smaller particles to enhance their solubility with the sodium silicate and distilled water. Then, the OSBA was mixed with sodium silicate and distilled water following the OSBA/Na_2_SiO_3_ ratios previously reported. Once the different alkali solutions were prepared, the next step consisted of weighing the precursor materials for the two types of geopolymers. In the control sample, the geopolymer was prepared with a constant amount of SSCS and Na_2_SiO_3_/NaOH with a weight ratio of 3 considering a 12 M solution. For the novel geopolymer, the SSCS and CH amounts also remained constant for the different families; however, the OSBA was added in increasing percentages (50%, 55%, 60%, and 65%) with different OSBA/Na_2_SiO_3_ weight ratios (1.7, 1.9, 2.2, and 2.4).

**Figure 1:**
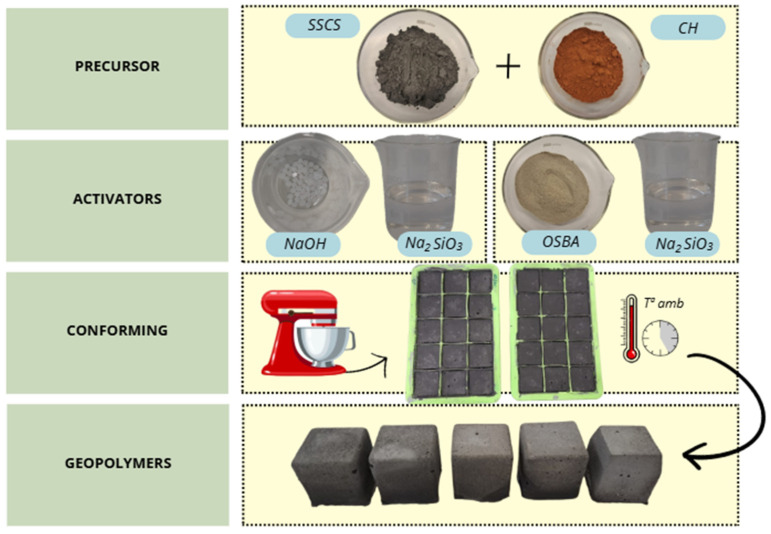
Schematic process preparation of geopolymers with SSCS, CH, OSBA, and Na_2_SiO_3_.

The same methodology for the conformation of the control and novel geopolymer families was followed. The weighed geopolymer pastes were poured into a prismatic mold with internal dimensions of 35 mm × 35 mm × 35 mm, with seven specimens per formulation. Once the geopolymer pastes were poured, they were vibrated for 15 s using a vibration table. As previously indicated, all samples were cured at an ambient temperature of 25 ± 2 °C for 72 h and were afterwards placed in an oven at 85 ± 5 for 90 h, then cured for 6 h again at an ambient temperature, with a total curing cycle of 7 days. Figure 1 schematically summarizes the process.

After the curing process was completed, samples were demolded and subsequently tested from chemical and physical standpoints. The physical and chemical characterizations consisted of determining, as follows: mass loss (UNE-EN 13581:2003) [71]; linear shrinkage (UNE-EN 13872:2004) [72]; capillary water absorption (UNE-EN 1015-18:2003) [73]; bulk density and open porosity (UNE-EN 1015-10:2000) [74]; and compressive strength (UNE-EN 1015-10:2020) [75]. Finally, Fourier transform infrared spectroscopy (FTIR) and scanning electron microscopy (SEM) were performed on the geopolymer samples with better physical and mechanical properties.

## 3. Results and Discussion

This chapter includes the section that describes the obtained results from the different tests described in the methodology chapter. The performed tests in this experimental research show partial conclusions that enabled us to assess the feasibility of this new binder formed by a combination of the waste valorization of chamotte, olive stone biomass bottom ash, and slate stone cutting sludge.

### 3.1. Physical and Chemical Characterization Test Results of the SSCS, OSBA, and CH

#### 3.1.1. Elemental Analysis

Samples of SSCS, OSBA, and CH were initially chemically characterized through the determination of carbon, hydrogen, and nitrogen. The elemental analysis of the mentioned by-products is shown in Table 2.

The elemental analysis of SSCS showed relatively low percentages of carbon (1.002%), hydrogen (0.336%), and nitrogen (0.103%). The percentage of organic carbon was considered as very insignificant and therefore would not negatively affect the geopolymerization process. The OSBA showed low hydrogen (2.760%) and nitrogen (0.102%) but a notable carbon value (6.150%). This value could have had its origin in the calcination process of the OSBA for this experiment, since the calcination process consists of the decomposition of potassium carbonate into potassium oxide and carbon dioxide at a calcination temperature of 950 °C. On the other hand, the chamotte showed negligible carbon (0.001%), hydrogen (0.015%), and nitrogen (0.001%) values. Since the selected raw materials did not contain relevant percentages of carbon that could lower the reaction process or affect the final geopolymer properties, they were considered suitable for the experimental program.

#### 3.1.2. X-Ray Fluorescence

The chemical composition of the SSCS, OSBA, and CH samples used for this research paper was obtained by X-ray fluorescence using the Bruker M4 Tornado spectrometer (Bruker: Billerica, MA, USA); the values, expressed in weight percentages, are shown in Table 3.

The values of SSCS reflect its suitability as a precursor due to its predominant composition of silica (51.85 wt.%) and a remarkable alumina value (23.25 wt.%). As reported in previous studies, slate stone has high percentages of silica and alumina, which enhances the reaction of geopolymerization with an alkali activator solution. The SiO_2_/Al_2_O_3_ ratio (2.44) is within the recommended range of 1 to 5 in which alkali gel formation occurs [79], contributing to the initial strength of geopolymer [59]. The SiO_2_/Al_2_O_3_ ratio of 2.44 also indicated the formation of PSS (poly(sialate-siloxo)), which can be produced during the alkali activation process, is a suitable material for cementitious applications, and has the capacity to encapsulate toxic wastes. The XRF also reflected some oxides, such as MgO (2.89%), and Fe_2_O_3_ (10.92%), which positively contributed to the final characteristics of the targeted geopolymer. Furthermore, the LOI of the SSCS was 1.5 wt.%. On the other hand, the chemical composition of CH indicated a pozzolanic activity [80], since most of the ceramic waste was composed of SiO_2_ (58.89%), Al_2_O_3_ (15.28%), and CaO (7.34%). Its calculated LOI was 3.34 wt.%. In the case of the OSBA, the K_2_O (27.48%) and CaO (24.22) content make it a potential alkaline activator for commercial activators such as NaOH, KOH, and Na_2_SiO_4_ [44]. Although these values may lead one to consider it a suitable activator, we anticipated that the bottom biomass ash would have less reactivity than fly biomass ash; therefore, during the experiment, it was expected that the CH would help to increase the reactivity of the bottom biomass.

#### 3.1.3. X-Ray Diffraction

The mineralogical composition of SSCS confirmed that the predominant oxides (SiO_2_ and Al_2_O3) appeared as quartz (Q) (SiO_2_) and muscovite (M) (KAl_2_(AlSi_3_O_10_)(OH)_2_) as well as K_2_O. The presence of quartz and muscovite is attributed to the sedimentary origin of the slate stone and its aluminosilicate-rich composition, which makes it suitable as a source material for geopolymerization.

Fe_2_O_3_ and MgO were present as clinochlore (Cl) ((AlSi_3_)O_10_(OH)_8_) and chamosite (Ch) ((Fe_5_Al)(AlSi_3_)(OH)_8_), both of which are common in metamorphic rocks, reflecting the geological processes that formed the slate. TiO_2_ appeared as rutile (R), which is a stable mineral phase under high-pressure and high-temperature conditions, further confirming the metamorphic history of the material.

**Figure 2:**
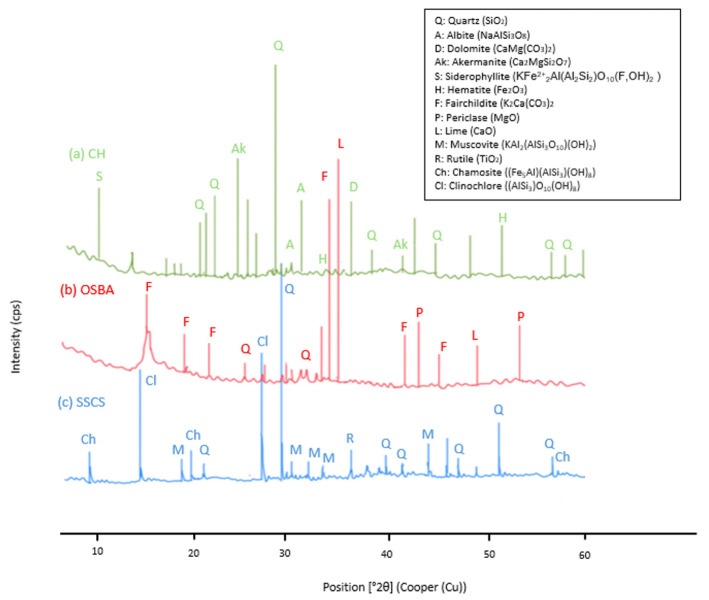
XRD pattern of SSCS, CH, and OSBA.

The diffraction pattern of the CH showed the crystalline phases, where, similar to SSCS, SiO_2_ and Al_2_O_3_, in the form of quartz (Q) (SiO_2_), were predominant. The presence of Fe_2_O_3_ as hematite (H), CaO as dolomite (D) (CaMg(CO_3_)_2_), and akermanite (Ak) (Ca_2_MgSiO_7_) can be explained by the calcination process of the chamotte, which transformed its raw clay minerals into stable crystalline phases. The OSBA mineralogical phases showed K_2_O and CaO as predominant compounds in the form of fairchildite (F) (K_2_Ca(CO_3_)_2_) and siltstone (L) (CaO). These phases are products of the high-temperature combustion of olive biomass, which results in carbonate-rich compounds such as fairchildite (F) (K_2_Ca(CO_3_)_2_), while the residual siltstone (L) (CaO) and traces of quartz (Q) (SiO_2_) and periclase (P) (MgO) reflect the original composition of the ash. Figure 2 shows the diffractograms of SSCS, OSBA, and CH. The identified crystalline phases highlight the suitability of these by-products for geopolymerization, as their mineralogical composition contributes essential aluminosilicate and alkaline components for the reaction mechanism.

#### 3.1.4. Particle Size Distribution of SSCS, CH, and OSBA

**Figure 3:**
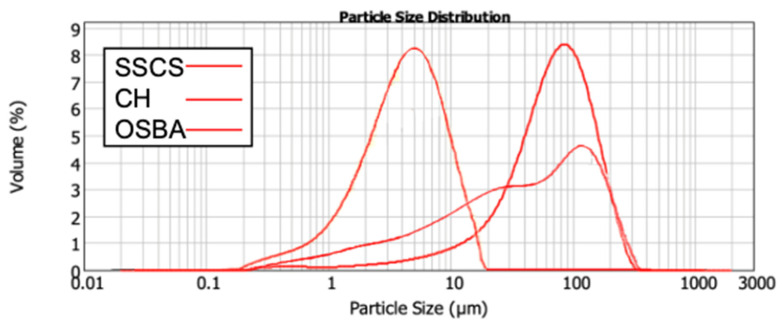
Graphical distribution of SSCS, OSBA, and CH.

Figure 3 shows the graphical distribution of SSCS, CH and OSBA particles, with particle sizes below 300 µm. The smaller particle size in SSCS influences the reactivity of the geopolymerization reaction due to the increased specific area (1.88 m^2^/g) [81]. The surface areas of CH and OSBA were lower (0.794 m^2^/g and 0.321 m^2^/g, respectively).

Table 4 shows the particle size distribution at D10, D50 and D90; it can be observed that, as fine powders, the three raw materials are of an adequate grain size, which enhances the mixing of materials and therefore the geopolymerization process.

#### 3.1.5. Density

The particle density of SSCS, OSBA, CH, and Na_2_SiO_3_ was determined using a pycnometer as per the UNE-EN-1097-7:2009 Standard [77]. The densities of SSCS, OSBA, CH and Na_2_SiO_3_ showed values close to 2.5 g/m^3^ (2.46, 2.74, 2.51 and 2.40 g/m^3^, respectively). The similarity of the values resulted in the better workability of the mixture and improved particle dispersion during the geopolymerization reaction. The density of the NaOH was 1.39 g/m^3^.

#### 3.1.6. pH Determination

pH plays a vital role in the kinetics of chemical reactions between the precursor materials (SSCS, CH) and the alkaline solution (OSBA, Na_2_SiO_3_); therefore, the pH alkalinity is an essential value for geopolymerization mechanisms and for obtaining noticeable mechanical characteristics. An average pH value of 8.62 was noted for the precursor raw material combination, and a pH of 13 for the alkali activation solution formed by the mixing of OSBA and Na_2_SiO_3_, indicated that the geopolymer formulation would achieve the pH value necessary for securing the geopolymerization process [20]. Table 5 shows the pH values of the precursors and alkaline activators.

#### 3.1.7. Fourier Transform Infrared Spectroscopy of the SSCS, CH, and OSBA

**Figure 4:**
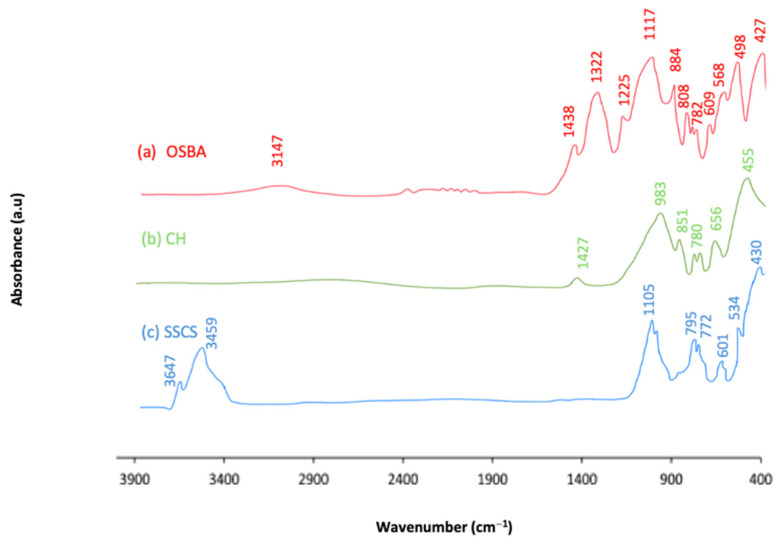
FTIR comparison of the SSCS, CH, and OSBA samples.

Attenuated total refraction Fourier transform infrared (ATR-FTIR) analysis was used to identify the functional groups of SSCS, CH, and OSBA, which are shown in Figure 4.

In the mining and olive raw materials, bands appeared at 3147 cm^−1^ for the OSBA, and at 3459 and 3647 cm^−1^ for the SSCS, which are associated with the stretching vibrations of the O-H group [82], indicating the presence of absorbed water. The stretching vibration of the C-O group, related to the carbonate content [18,83,84], was demonstrated in the peaks at 1438 cm^−1^ and 1427 cm^−1^ for the OSBA and CH, respectively. The bands attributed to the stretching vibrations of Si-O-T bonds, typical in materials rich in silica (SiO_2_), were identified with wavenumbers between 983 and 971 cm^−1^ for the SSCS and CH, due to their rich silica content, as previously reported in Table 3. The bands appearing at around 800 cm^−1^ for the OSBA and SSCS (884, 808 and 795 cm^−1^, respectively) are associated with carbonate partitioning due to environmental exposure. The bands appearing at 782, 780 and 772 cm^−1^ indicated symmetrical Si-O-Si stretch bending in the OSBA, CH and SSCS. The bands appearing between 656 and 568 cm^−1^ were related to the bending vibration due to the presence of quartz [83,84] in the analyzed SSCS, OSBA, and CH samples. The presence of quartz in the different raw materials was corroborated in the mineralogical composition determination, shown in Section 3.1.3. Finally, bands at lower wavenumbers were related to the bending vibration of Si-O (between 534 and 427 cm^−1^) [84]. Table 6 shows the characteristic FTIR peaks of OSBA, CH, and SSCS.

### 3.2. Physical and Mechanical Characterization Test Results of the Novel Geopolymers Using SSCS, OSBA, and CH

#### 3.2.1. Determination of Mass Loss

**Figure 5:**
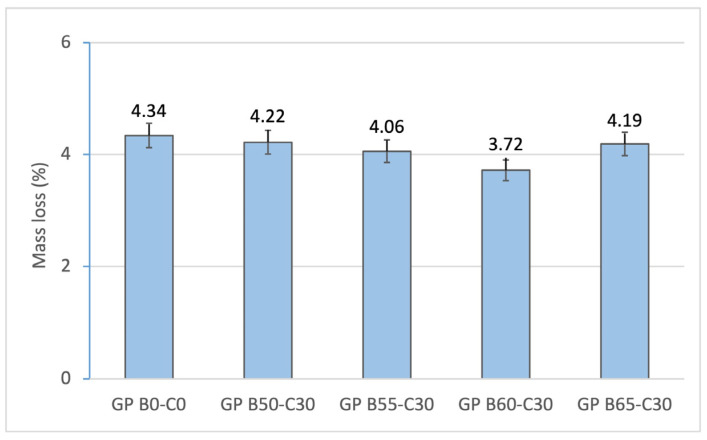
Mass loss of control geopolymer and novel geopolymers prepared with SSCS, CH, OSBA, and Na_2_SiO_3_.

The determination of the mass loss of the geopolymers after the curing process is associated with the loss of free water in the mortars. Figure 5 shows the average values of the different geopolymer samples prepared for the experiment. The control geopolymer GP B0-C0, prepared with the lowest amount of water, shows a mass loss of 4.14%. The geopolymers using OSBA as the alkaline activator (GP B50-C30, GP B55-C30, GP B60-C30, GP B65-C30) show slightly lower mass loss ratios (4.22, 4.06, 3.72, 4.19%, respectively). This phenomenon is associated with the increase in reactivity experienced by the mortar with the addition of CH to the mix and with the increase in the specific area of the OSBA particles. The best results were obtained for GP B60-C30 (3.72%), containing 60% OSBA. The increase in the mass loss percentage of GP B65-C30 is related to the increase in unreacted OSBA particles produced by a low OSBA/Na_2_SiO_3_ ratio (2.4) and by the increase in the liquid/agglutinant ratio (0.98).

#### 3.2.2. Determination of Linear Shrinkage

**Figure 6:**
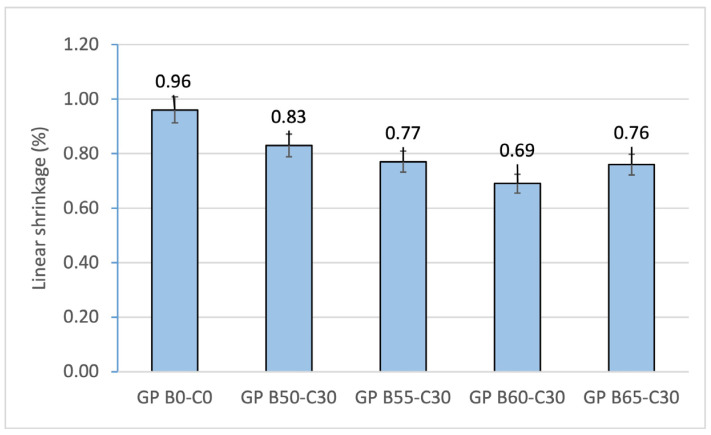
Linear shrinkage of control geopolymer and novel geopolymers prepared with SSCS, CH, OSBA, and Na_2_SiO_3_.

The results obtained for linear shrinkage (Figure 6), related to mass loss, show a similar trend. All the obtained linear shrinkage values were less than 1%. The control geopolymer GP B0-C0 presented the highest linear shrinkage value, which is related to the packing of the alkaline activator particles. The OSBA-activated geopolymer families show lower values (between 0.69 (GP B50-30) and 0.83% (GP B60-C30)). This decrease is associated with the morphology of the OSBA particles, which decreased the porosity of the mortar during the curing process [85]. The decrease in the percentage of linear shrinkage in GP B60-C30 is associated with the increased reactivity of the geopolymer with the OSBA/Na_2_SiO_3_ ratio of 1.0 and the stabilization of the specimen volume.

#### 3.2.3. Capillary Water Absorption and Cold-Water Absorption Determination

**Figure 7:**
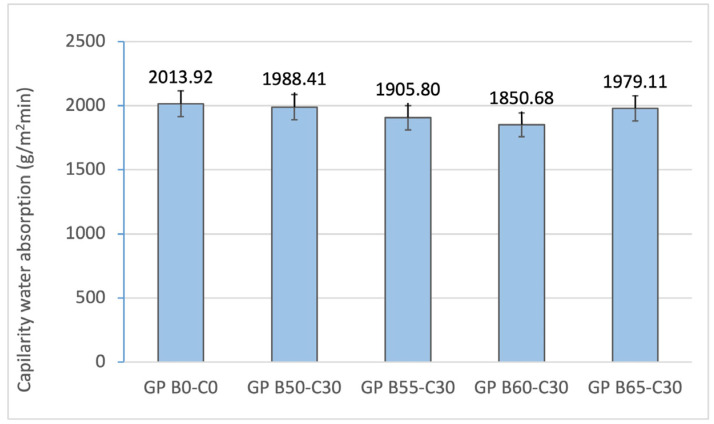
Capillary water absorption of control geopolymer and novel geopolymers prepared with SSCS, CH, OSBA, and Na_2_SiO_3_.

**Figure 8:**
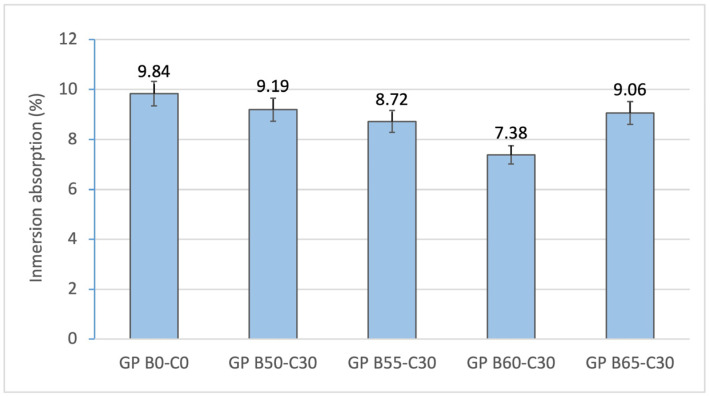
Cold-water absorption of control geopolymer and novel geopolymers prepared with SSCS, CH, OSBA, and Na_2_SiO_3_.

Figure 7 and Figure 8 show how increasing the liquid/binder ratio from 0.16 (GP B0-C0) to 0.95 (GP B60-C30) influences the decreasing values of capillary (2013.92 to 1850.68 g/m^2^min) and immersion (9.84 to 6.38%) absorption. The reduction in water absorption is associated with the increased reactivity of the geopolymer mortar and the densification of the matrix with higher liquid/binder ratios. The increase in the absorption percentage in GP B65-C30 is related to the excess of alkaline activator in the geopolymer.

#### 3.2.4. Determination of Open Porosity

**Figure 9:**
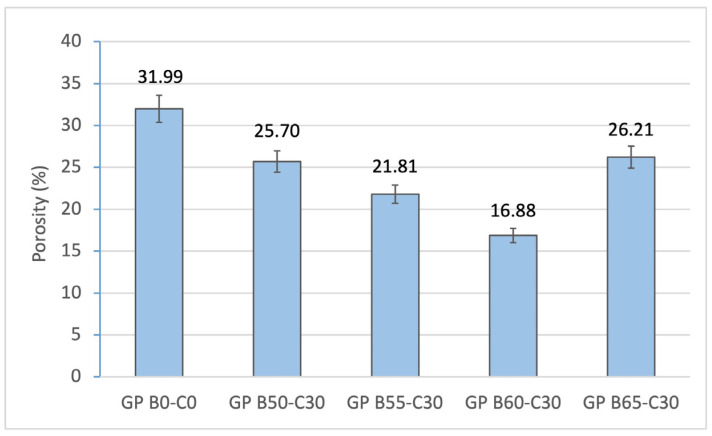
Open porosity of control geopolymer and novel geopolymers prepared with SSCS, CH, OSBA, and Na_2_SiO_3_.

Figure 9 shows the water absorption is directly associated with the porosity of the geopolymers. It can be seen that increasing the OSBA/Na_2_SiO_3_ ratio from 0.91 (GP B50-C30) to 0.95 (GP B60-C30) decreases the porosity values from 25.70 to 16.88% compared to the value obtained for the control geopolymer (GP B0-C0) (31.99%). The lower porosity of the more reactive geopolymer families (GP B55-C30 and GP B60-C30) is related to the densification of the matrix, where the size and number of pores decreases.

#### 3.2.5. Determination of Bulk Density

**Figure 10:**
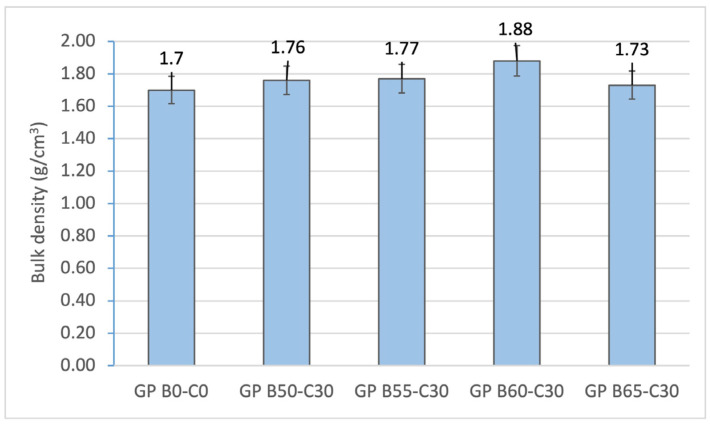
Bulk density of control geopolymer and novel geopolymers prepared with SSCS, CH, OSBA, and Na_2_SiO_3_.

The bulk density of the geopolymers is inversely proportional to the porosity (Figure 10). The geopolymers containing GP B50-C30, GP B55-C30, and GP B60-C30 show a higher density (1.76, 1.77, 1.88 g/cm^3^, respectively) due to the lower porosity (25.7, 21.8 and 16.9%, respectively) obtained in the matrixes by increasing the OSBA/Na_2_SiO_3_ ratio from 1.69 to 2.2. The control geopolymer GP B0-C0 shows a lower density of 1.7, which is associated with the lower density of NaOH (1.39 g/mL) and to the absence of CH, which increases the reactivity and densifies the matrix.

#### 3.2.6. Compression Strength Test Results

**Figure 11:**
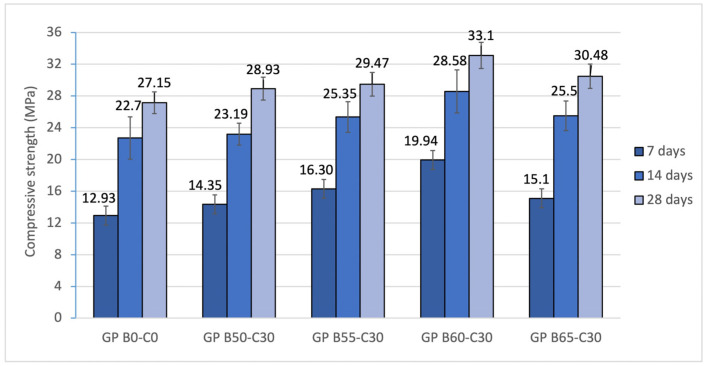
Compressive strength of control geopolymer and novel geopolymers prepared with SSCS, CH, OSBA, and Na_2_SiO_3_.

The mechanical strength of the 5 geopolymer families was studied at 7, 14 and 28 days (Figure 11). The results obtained show how the curing time influences the strength values. It was observed that during the first 7 days of curing, the geopolymers achieved about 50% of their total strength. The increase in compressive strength as a function of curing time is associated with the geopolymerization reaction and the gelation of the mortar [21]. Partial substitution of OSBA with Na_2_SiO_3_ resulted in an increase in strength from 28.93 (GP B50-C30) to 33.10 MPa (GP B60-C30) at 28 days. The control geopolymer (GP B0-C0) showed slightly lower values (27.15 MPa). The strength values in the GP B50-C30, GP B55-C30, GP B60-C30 and GP B65-C30 families are related to the increased reactivity of the mortar by using OSBA as an alkaline activator and adding CH (30%) to increase the aluminosilicate content of the geopolymer blends. The more reactive mortars produced denser mortars with smaller porous cavities. GP B60-C30 and GP B65-C30 showed the best strength results; however, a decrease in the value from 33.1 to 30.48 MPa was observed when increasing the OSBA content from 60 to 65%, respectively, and decreasing the Na_2_SiO_3_ content. This is the direct consequence of an excess of OSBA in the matrix that is related to the increase in the Na/Si ratio. This was demonstrated by the presence of Na^+^ ions, which promoted the formation of a uniform matrix as a consequence of the movement of the silicate species connecting the particles of the reaction products [84].

On the other hand, GP B50-C30 and GP B55-C30, with lower OSBA/Na_2_SiO_3_ ratios (1.7 and 1.9, respectively) showed lower strength values at 28 days (28.93 and 29.97 MPa, respectively) as a consequence of the lower reactivity of the alkaline activator at the abovementioned ratios, which produced less dense matrixes.

#### 3.2.7. Fourier Transform Infrared Spectroscopy of the Control and Novel Geopolymers

**Figure 12:**
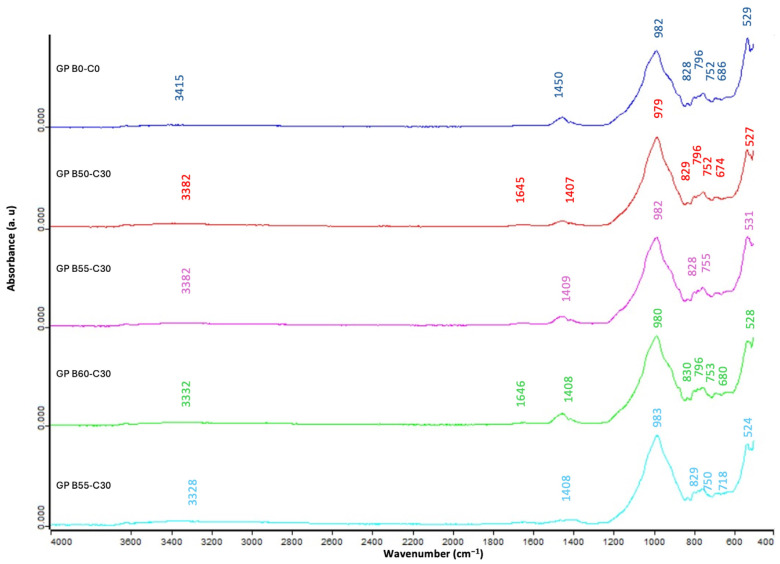
FTIR comparison of the control geopolymer and novel geopolymers prepared with SSCS, CH, OSBA, and Na_2_SiO_3_.

The FTIR analysis was used to determine the chemical bonds, nature of bonding, and to understand the mechanisms of the geopolymerization process for the control geopolymer and target geopolymer samples. The bands located between 3415 cm^−1^ and 3328 cm^−1^ were assigned to the stretching vibration of the OH bond [85]. The bands between 1650 cm^−1^ and 1640 cm^−1^ were due to the bending vibration of H-O-H., in turn, caused by the presence of water molecules in the alkali-activated binder pores [86]. The behavior observed in these bands is relevant for long-term stability, as the amount and mobility of the retained water can influence the susceptibility of the material to deterioration by freeze–thaw cycles, as well as the resistance to prolonged environmental humidity. In general, a lower intensity in these bands is associated with a lower amount of physically adsorbed water and, therefore, the higher durability of the geopolymer. Figure 12 represents the graphical comparison of the different FTIR patterns, where it can be observed that increasing the percentage of OSBA led to a decrease in the wavenumbers of peaks associated with the stretching vibration of the OH bond. This could be associated with the higher degree of geopolymerization reaction and the greater density of the geopolymer material, and, therefore, fewer water molecules on the geopolymer surface. The asymmetric stretching vibration of the O-C-O peaks were in the range of 1432 cm^−1^ and 1412 cm^−1^, indicating the presence of carbonates (CO^3^)^2−^ formed during the calcination process [31]. These peaks in the geopolymer samples conformed with different percentages of OSBA, indicating that carbonates had formed because of the calcination process, which consisted of the decomposition of potassium carbonate into potassium oxide and carbon dioxide. The asymmetric stretching vibration of Si-O-T was shown in peaks between 1408 cm^−1^ and 1407 cm^−1^ for the targeted geopolymer samples, reflecting the geopolymerization reaction [87,88,89]. Wavenumbers in this range show the typical gradual progress for gel formation during the geopolymerization process; it is also an indicator of remarkable compressive strength as a direct consequence of the high concentration of Si-O-Al bonds. The bands at 796 cm^−1^ and 795 cm^−1^ referred to the bending symmetric stretching vibration Si-O-Si bonds in the network of silica non reacted [90]. The wavenumber range of 674 cm^−1^ and 718 cm^−1^, and the band range of 527 cm^−1^ and 524 cm^−1^, refer, respectively, to the pattern of bending vibrations of Si-O-Si and Si-O bonds of quartz [80], providing information about the incomplete dissolution of quartz phases, as shown in the XRD analysis. The presence of these residual phases can negatively impact long-term chemical stability in aggressive environments; however, their contribution is less significant when the material is used in controlled or moderately aggressive environments. Table 7 summarizes the characteristic absorption peaks of the FTIR spectra of the control geopolymer and novel geopolymers.

#### 3.2.8. X-Ray Diffraction of the Control and Novel Geopolymers

**Figure 13:**
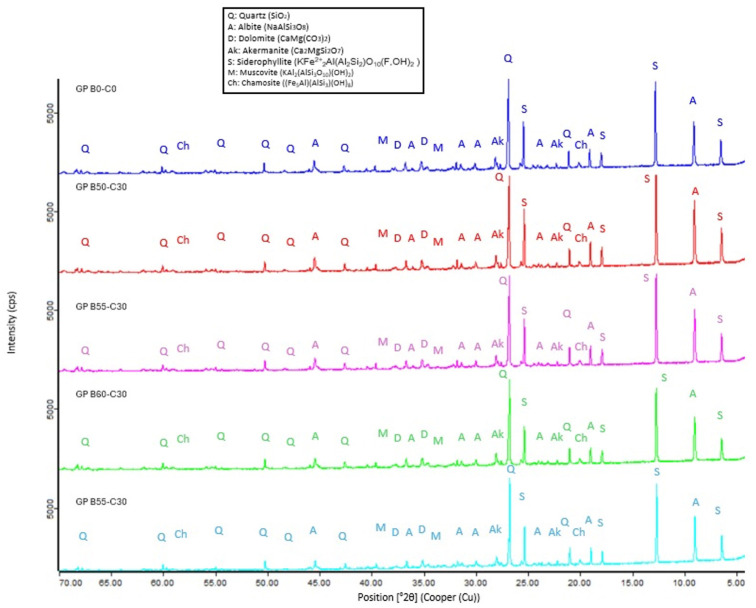
XRD pattern of control geopolymer and novel geopolymers.

The analysis of the mineralogical composition of the geopolymers (Figure 13) shows that the crystalline phases appearing in the precursors (SSCS and CH) and in the alkaline activator (OSBA) (Figure 2) appear mostly in the formed geopolymers. Aluminosilicates in the form of quartz (Q) (SiO_2_) and muscovite (M) (KAl_2_(AlSi_3_O_10_)(OH)_2_) prevail. The presence of quartz (Q) suggests that the geopolymerization reaction did not fully occur. The intensity of the albite (A) (NaAlSi_3_O_8_) peaks decreased with the increasing OSBA/Na_2_SiO_3_ ratio, which is related to a higher degree of gel formation [87].

#### 3.2.9. Scanning Electron Microscopy (SEM) and Energy-Dispersive X-Ray Spectroscopy (EDX) Analysis of Control Geopolymer and Novel Geopolymers

**Figure 14:**
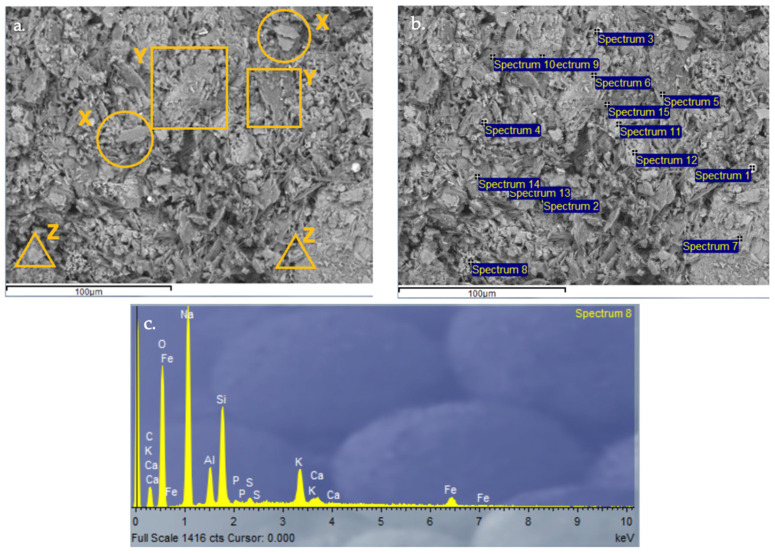
SEM microscopy of control geopolymer GP B0-C0 sample: (**a**) Entire area of SEM secondary 3000× image (**b**) Entire area of SEM retro-dispersed 2000× image with selection of 15 points of spectrum; and (**c**) EDX Analysis of spectrum 8.

**Figure 15:**
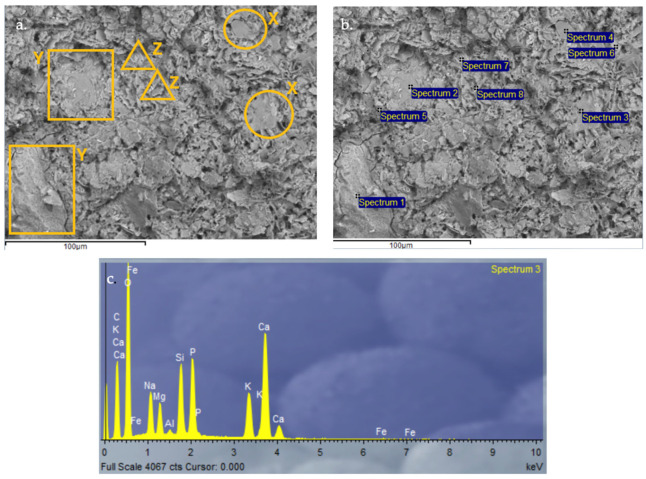
SEM microscopy of geopolymer GP B50-C30 sample: (**a**) Entire area of SEM secondary 3000×; (**b**) Entire area of SEM retro-dispersed 2000× image with selection of 8 points of spectrum; and (**c**) EDX Analysis of spectrum 3.

**Figure 16:**
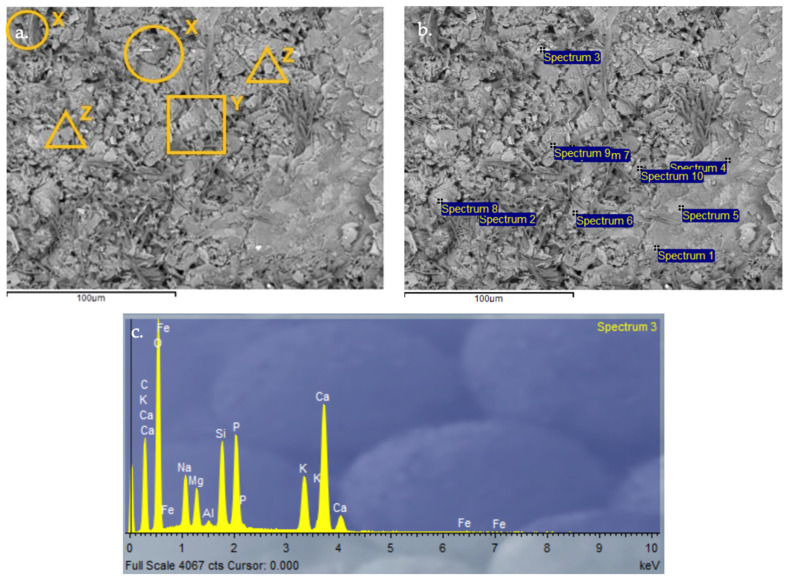
SEM microscopy of geopolymer GP B55-C30 sample: (**a**) Entire area of SEM secondary 3000× image; (**b**) Entire area of SEM retro-dispersed 2000× image with selection of 10 points of spectrum; and (**c**) EDX Analysis of spectrum 3.

**Figure 17:**
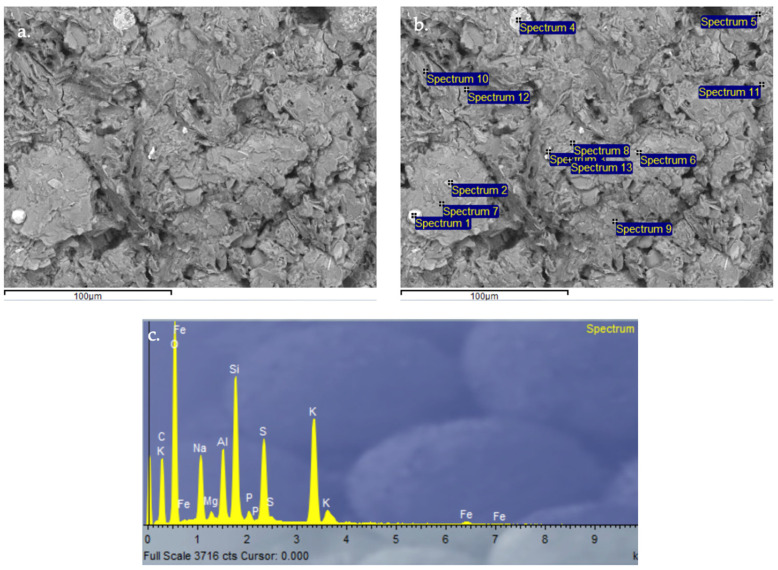
SEM microscopy of geopolymer GP B60-C30 sample: (**a**) Entire area of SEM secondary 3000× image; (**b**) Entire area of SEM retro-dispersed 2000× image with selection of 13 points of spectrum; and (**c**) EDX Analysis of spectrum 8.

**Figure 18:**
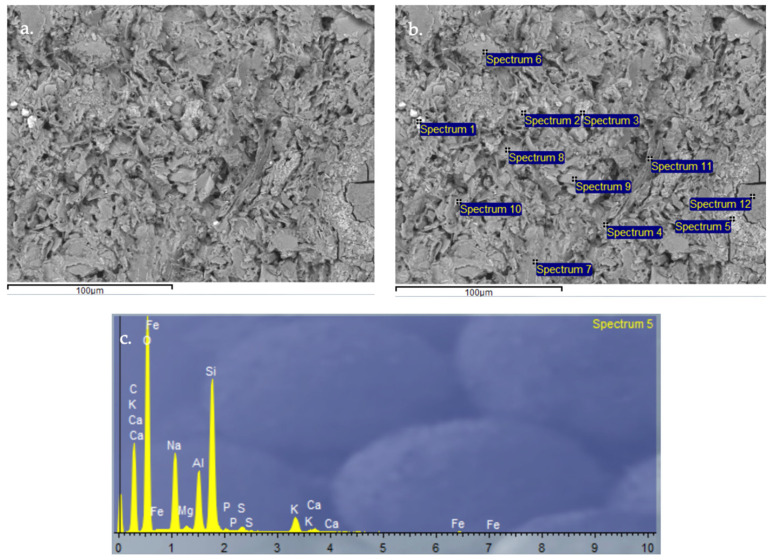
SEM microscopy of geopolymer GP B65-C30 sample: (**a**) Entire area of SEM secondary 3000× image; (**b**) Entire area of SEM retro-dispersed 2000× image with selection of 12 points of spectrum; and (**c**) EDX Analysis of spectrum 5.

After 28 days of curing, all geopolymers were analyzed by SEM-EDX. The microscopy results verify the obtained values for absorption, density, porosity, and compressive strength. The control geopolymer GP B0-C0 (Figure 14) shows a densified and homogeneous microstructure associated with alkaline gel formation. The EDX analysis showed that the aluminosilicate content is homogeneously distributed throughout the matrix. GP B50-C30 (Figure 15) and GP B55-C30 (Figure 16) show a less homogeneous and densified porous structure because fewer reaction products were formed due to the lack of reactivity of the mortar with a lower proportion of OSBA (Y) (50 and 55%, respectively). The presence of unreacted OSBA particles, identified in the SEM, demonstrated the incomplete dissolution of the precursors in the alkaline solution, limiting the formation of aluminosilicate gels. This resulted in a microstructurally weak matrix, with higher porosity and lower internal cohesion, which explains the observed decrease in compressive strength and lower bulk density values. The traces of calcium carbonate (CaCO_3_) that appear are associated with the deficient dissolution of CH (Z) and OSBA [91,92], which reinforced the correlation between the lack of complete reaction of the precursors and the reduction in the mechanical properties. On the contrary, GP B60-C30 (Figure 17) shows a matrix composed of homogeneous and more compact particles. It is associated with the complete dissolution of the precursors (SSCS (X) and CH) with the alkaline activator (OSBA and Na_2_SiO_3_). The SEM analysis confirmed the absence of unreacted particles, and the EDX verified the absence of Ca inclusions, indicating a complete reaction of the precursors and activators. This indicates the formation of a well-structured crystal lattice [93]; the high densification of the matrix resulted in higher compressive strength and improved mechanical properties. On the other hand, in GP B65-C30 (Figure 18) an excess of alkaline activator produced a matrix in which unreacted particles can be observed due to the oversaturation of Si in the alkaline solution. This inhibited efficient polymerization, as evidenced by the areas of low cohesion and unreacted particles observed in the SEM. This behavior negatively affected the integrity of the matrix, explaining the reduced compressive strength values.

## 4. Conclusions

Once the physicochemical characterization of the three different raw materials was completed, and the physical and mechanical tests of the different families of the conformed geopolymers were fully developed and technically interpreted, a list of conclusions was obtained. The aim of this study was to determine the suitability of a combination of mining, ceramics, and olive industry by-products as a sustainable potential binder material to be used as an alternative to ordinary Portland cement.

The following conclusions were drawn from this study:

- The particle size distribution of slate stone cutting sludges and olive stone bottom ash shows that both by-products are composed of finer particles without the need for crushing operations. On the contrary, crushing and griding was required for the chamotte to yield a particle size of 250 μm, sieved. However, during the mixing preparation of the source of the aluminosilicates—chamotte and slate stone cutting sludge—and the alkali activation composed of the olive stone bottom ash and sodium silicates, no homogenization problems arose. The small particle size distributions ensured the reactivity between the particles and, therefore, the geopolymerization process;
- The elemental analysis of the studied raw materials indicated negligible values of carbon, hydrogen, and nitrogen. Therefore, it was foreseen that the geopolymerization process would not be affected or jeopardized;
- The chemical composition analysis of the SSCS showed remarkable percentages of silica and alumina, 51.85 and 23.25 wt.%, respectively. The XRF of the CH indicated a pozzolanic activity due to its majority composition of silica (58.89 wt.%) and low percentage of alumina (15.28 wt.%). The combination of both raw materials, due to the percentages of silica and alumina, became the potential source of aluminosilicates for the experiment;
- The analysis of the chemical composition of the olive stone biomass bottom ash indicated that it was mainly composed of potassium oxide and calcium oxide, with percentages of 27.48 and 24.22 wt.%, respectively. These values indicated that the OSBA was suitable for use as an activator. However, because the bottom biomass ash is less reactive than fly ash, the CH was used to increase its reactivity;
- The optimal geopolymer formula was 60% olive biomass bottom ash and 30% chamotte, with an OSBA/Na_2_SiO_3_ weight ratio of 2.2. The highest value of compressive strength was 33.1 MPa, which was obtained after 28 days of curing;
- The physical tests on the different geopolymer families showed that the variation in the amounts of OSBA in the alkali solution plays an important role in the final features of the novel geopolymers. It was observed that mass loss, linear shrinkage, immersion, capillary, and open porosity percentages decreased with lower ratios of OSBA/Na2SiO3 alkali solution, with ratios up to 2.2, and then increased;
- The bulk density and compressive strength values showed that when the OSBA/Na_2_SiO_3_ ratio increased, both values increased up to a maximum value of 2.2. From this ratio, compressive strength and density tended to decrease due to an excess of OSBA that did not react, resulting in a retardation of the geopolymerization process, leading to a geopolymer that was not fully reacted;
- From an environmental standpoint, this potential binder can provide a solution to the uncontrolled accumulation of slate stone cutting sludge at mining sites as well as the accumulation of chamotte in landfills;
- For the specific case of bottom ash from olive oil production (OSBA), its use as a potential alkali activator solution in combination with Na_2_SiO_3_ provides the opportunity to reduce the consumption of commercial activators. Unlike fly ash from the olive oil industry, which is mainly used as a fertilizer due its potassium content, the bottom ash is normally disposed of without further use. Therefore, its use in the manufacturing of new binders for construction materials will mitigate its accumulation;
- The results obtained from the physical, chemical, mechanical and microstructural characterizations will allow the process to be extrapolated to industrial applications. To this end, the manufacturing process of the geopolymeric mortar would be scalable through the arrangement of the materials in hoppers and their subsequent mixing in industrial mixers with the prior preparation of the alkaline activator in another mixer. The low curing temperatures required make the scalability of the process perfectly viable in industrial applications.

Based on the analysis on the previous results and the conclusions drawn, the feasibility of slate stone cutting sludge (SSCS) and chamotte (CH) as precursors, and olive stone bottom ash (OSBA) in combination with Na_2_SiO_3_ as an alkaline activator solution, was demonstrated. The best mechanical properties were obtained using a weight ratio of 2.2, which showed a remarkable compressive strength value of 33.1 MPa after 28 days of curing. The mechanical strength value of 33.1 MPa is higher than that required for construction mortar in the M20 category (20 MPa as per UNE-EN 1015-11:2020), which means that the geopolymer derived in the study is a potential sustainable material for use in the construction sector.

## Figures and Tables

**Table 1:** Relationship of geopolymer families for control geopolymer and novel geopolymer samples with SSCS, CH, OSBA, and Na_2_SiO_3._

Sample Series Code	Solid (g)	CH (%)	NaOH 12 M (%)	OSBA (%)	Na_2_SiO_3_/NaOH Weight Ratio	OSBA/Na_2_SiO_3_ Weight Ratio	Liquid/Solid Weight Ratio
GP B0-C30	240	0	15	-	3.00	-	0.16
GP B50-C30	240	30	-	50	-	1.7	0.91
GP B55-C30	240	30	-	55	-	1.9	0.93
GP B60-C30	240	30	-	60	-	2.2	0.95
GP B65-C30	240	30	-	65	-	2.4	0.98

**Table 2:** Elemental analysis of SSCS, OSBA, and CH.

Sample	Nitrogen, %	Carbon, %	Hydrogen, %
SSCS ^1^	0.103 ± 0.003	1.002 ± 0.057	0.336 ± 0.002
OSBA ^2^	0.102 ± 0.003	6.150 ± 0.057	2.760 ± 0.002
CH ^3^	0.001 ± 0.003	0.446 ± 0.057	0.015 ± 0.002

^1^ Slate stone cutting sludge; ^2^ Olive stone bottom ash; ^3^ Chamotte.

**Table 3:** X-Ray fluorescence of SSCS, OSBA, and CH.

Compound	SSCS, wt.%	OSBA, wt.%	CH, wt.%
SiO_2_	51.85	4.12	58.89
Al_2_O_3_	23.25	0.770	15.28
CaO	0.399	24.22	7.34
Fe_2_O_3_	10.92	0.94	7.45
K_2_O	4.42	27.48	5.01
MgO	2.89	3.82	2.43
TiO_2_	1.24	0.060	0.87
Na_2_O	1.16	0.761	0.42
SO_3_	0.458	0.125	0.83
P_2_O_5_	0.25	1.85	0.16
LOI ^1^	5.01	20.25	3.43

^1^ Loss on ignition.

**Table 4:** Particle size distribution of SSCS, OSBA, and CH.

Distribution (μm)	D10	D50	D90
SSCS ^1^	1.602	5.179	11.991
OSBA ^2^	17.580	70.618	51.404
CH ^3^	3.354	51.404	239.542

^1^ Slate stone cutting sludge; ^2^ Olive stone bottom ash; ^3^ Chamotte.

**Table 5:** pH determination of SSCS, OSBA, CH, NaOH, and Na_2_SiO_3_.

Raw Material	pH Average	Temp. (°)
SSCS ^1^	8.58	24.8
OSBA ^2^	11.75	24.5
CH ^3^	8.66	24.4
NaOH ^4^	13.7	24.5
Na_2_SiO_3_^5^	13.0	24.6

^1^ Slate stone cutting sludge; ^2^ Olive stone bottom ash; ^3^ Chamotte; ^4^ sodium hydroxide; ^5^ sodium silicate.

**Table 6:** Characteristic absorption peaks of the FTIR spectra for the OSBA, CH, and SSCS samples.

Function Group	Wavenumber Range (cm^−1^)		FTIR Peaks (cm^−1^)		
Raw Material		OSBA	CH	SSCS	Reference
Stretching vibration O-H	3647–3147	3147	-	3647, 3459	[80]
Asymmetric stretching vibration C-O	1438–1427	1438	1427	-	[18,81,82]
Asymmetric stretching vibration Si-O-T	983–971	-	983	971	[81,82]
C-O bond vibrations in carbonate groups	884–795	884, 808	-	795	[81,82]
Bending symmetric stretching vibration Si-O-Si	782–772	782	780	772	[81,82]
Bending vibration in quartz	656–568	609, 568	656	601	[82]
Bending vibration Si-O	534–427	498, 427	455	534, 430	[82]

**Table 7:** Characteristic absorption peaks of the FTIR spectra for the GPB0-C0, GPB50-C30, GPB55-C30, GPB60-C30, GPB65-C30 samples, function groups, and wavenumber ranges.

Function Group	Wavenumber Range (cm^−1^)	FTIR Peaks (cm^−1^)				
Raw material		GPB0-C0	GPB50-C30	GPB55-C30	GPB60-C30	GPB65-C30
Stretching vibration O-H	3300–3700	3415	3382	3382	3332	3328
Bending vibration H-O-H	1622–1658	1650	1645	1646	1646	1640
Asymmetric stretching vibration O-C-O	1432–1412	1450	1407	1409	1408	1408
Asymmetric stretching vibration Si-O-T	1230–900	982	979	982	980	983
C-O bond vibrations in carbonate groups	870–810	828	829	828	830	829
Bending symmetric stretching vibration Si-O-Si	800–700	796	796	795	796	795
Bending vibration in quartz Si-O-Si	690–600	686	674	673	680	718
Bending vibration in Si-O in quartz	400–500	529	527	531	528	524

## Data Availability

The original contributions presented in this study are included in the article. Further inquiries can be directed to the corresponding author.

## Abbreviations

The following abbreviations are used in this manuscript:

SSCS	Slate stone cutting sludge
OSBA	Olive stone bottom ash
TLA	Three-letter acronym
CH	Chamotte
